# Measuring Spatial Accessibility of Health Care Providers – Introduction of a Variable Distance Decay Function within the Floating Catchment Area (FCA) Method

**DOI:** 10.1371/journal.pone.0159148

**Published:** 2016-07-08

**Authors:** Jan Bauer, David A. Groneberg

**Affiliations:** Institute of Occupational, Social and Environmental Medicine, Goethe University, Frankfurt/Main, Hessen, Germany; Brighton and Sussex Medical School, UNITED KINGDOM

## Abstract

We integrated recent improvements within the floating catchment area (FCA) method family into an integrated ‘iFCA`method. Within this method we focused on the distance decay function and its parameter. So far only distance decay functions with constant parameters have been applied. Therefore, we developed a variable distance decay function to be used within the FCA method. We were able to replace the impedance coefficient *β* by readily available distribution parameter (i.e. median and standard deviation (SD)) within a logistic based distance decay function. Hence, the function is shaped individually for every single population location by the median and SD of all population-to-provider distances within a global catchment size. Theoretical application of the variable distance decay function showed conceptually sound results. Furthermore, the existence of effective variable catchment sizes defined by the asymptotic approach to zero of the distance decay function was revealed, satisfying the need for variable catchment sizes. The application of the iFCA method within an urban case study in Berlin (Germany) confirmed the theoretical fit of the suggested method. In summary, we introduced for the first time, a variable distance decay function within an integrated FCA method. This function accounts for individual travel behaviors determined by the distribution of providers. Additionally, the function inherits effective variable catchment sizes and therefore obviates the need for determining variable catchment sizes separately.

## Introduction

Adequate access to health care providers is crucial for any health care system. However, there is still confusion of what is actually meant by “access”. Access is a multidimensional construct consisting on a variety of social, financial, geographical and personal factors [1]. As outlined in the World Health Report from 2010 it is estimated that a high proportion of the world’s poor population has no access to health services because they can’t afford it [2]. This being said, the world is a long way from universal coverage especially in low income countries.

According to Joseph et al. healthcare access can be potential (i.e. possibility of access) and/or revealed (i.e. actual use of access) [3]. Both can further be divided into spatial factors (e.g. geographic barriers) and non-spatial factors (e.g. social barriers). Therefore, barriers can impede potential access to become revealed access. Five barriers have been described by Penchansky et al.: availability, accessibility, accommodation, affordability and acceptability [4]. Availability (number of health care providers) and accessibility (the distance/time from demand to supply of health care) are commonly combined and referred to as “spatial accessibility” [5]. In this paper we focused on the measurement of potential spatial accessibility (SA).

Gravity models, as suggested by Joseph et al., are more sophisticated measures of spatial accessibility than simple population-to-provider ratios (PPR) [3]. However gravity models still have limitations due to difficulties choosing an appropriate distance decay function with the appropriate impedance coefficient *β* [5]. Due to these limitations, Luo et al. developed the two step floating catchment area (2SFCA) method, which is a special case of a gravity model based on spatial decomposition [6,7]. This 2SFCA method has been subject to improvement several times [8,9]. All derived methods are now part of the floating catchment area (FCA) family. Based on the 2SFCA method, we aimed at improving the FCA method by several factors. First, we present an integrated FCA method by combining recent improvements on the FCA methods. Second, we introduce a variable distance decay function dependent on population-to-provider distances distribution (median and standard deviation) rather than a constant *β* parameter as in earlier approaches. Third, we show that this variable distance decay function inherits effective variable catchment sizes. Finally we present the proposed method in a case study of the metropolitan area of Berlin, Germany.

## Material and Methods

### Earlier approaches

Since all FCA methods are based on the 2SFCA method, we will briefly demonstrate its principles: The 2SFCA method keeps the advantages of a gravity model while it’s easier to interpret as it represents a derived form of a PPR. As the name suggests, two steps have to be performed:

Step 1: For each provider location *y*, look up all population locations *x* that are within a predefined global catchment size *C*_*glob*_ (time/distance) from location *y*. Sum up all population sizes (*P*_*x*_) within that catchment area. Compute the provider-to-population ratio (*PPR*_*y*_) within that catchment *y*, where *S*_*y*_ is the capacity of provider location *y* (e.g. number of providers or number of hospital beds):

$${PPR}_{y}=\frac{S_{y}}{\sum_{x\in \left(d_{xy}\leq C_{glob}\right)}P_{x}}$$ (1)

Step 2: For each population location *x*, look up all provider locations *y* that are within the catchment from location *x*. Sum up all *PPR*_*y*_ for the catchment area to calculate the spatial Accessibility Index (*AI*_*x*_) at location *x*:

$${AI}_{x}\;=\sum_{y\in (d_{xy}\leq C_{glob})}\;{PPR}_{y}$$ (2)

Despite its superiority to simpler measures of spatial accessibility, the 2SFCA method has three shortcomings: 1) catchment sizes are fixed, 2) no distance decay function is applied within a catchment and 3) omission of competition [8,10–12]. In order to address these shortcomings, the 2SFCA method has been improved and modified several times since its publication in 2003. Regarding the distance decay, both stepwise and continuous approaches have been applied (see enhanced (E)2SFCA method or kernel density function (KD)2SFCA method) [8,13,14]. However, different decay functions have been used including the Gaussian function and gravity functions [11]. Besides the selection of the function itself, the choice of the appropriate parameter, namely the impedance coefficient *β* further increases uncertainty [15]. As pointed out by Wang, the *β* parameter itself should rather be a variable instead of a constant [16]. Regarding catchment sizes, recent literature suggests variable catchment sizes rather than fixed catchment sizes (see variable (V)2SFCA method or enhanced variable (EV)2SFCA method) [17–19]. Regarding competition, the 3SFCA method included competition by accounting for the number of competitors within a catchment [20]. Furthermore, the Huff Model was introduced to the FCA methods to account for competition [21,22]. A more detailed review of earlier FCA methods including their shortcomings are provided within supporting information file S1 Appendix.

### Integration of recent improvements

We integrated suggested improvements on the FCA method outlined above. This integrated FCA method ‘iFCA’ can be displayed with the following formula.

$${AI}_{x}\;=\sum_{y\in (d_{xy}\leq C_{x})}\;\frac{S_{y}*f_{adj}\left(d_{xy}\right)*f_{con}\left(d_{xy}\right)}{\sum_{x\in (d_{xy}\leq C_{x})}P_{x}*f_{adj}(d_{xy})*{Prob}_{demand}}$$ (3)

where *AI*_*x*_ is the potential spatial accessibility index at location *x*, *S*_*y*_ is the capacity of provider at location *y*, *P*_*x*_ is the population size at location *x*, *f*_*adj*_*(d*_*xy*_*)* is the adjusted and *f*_*con*_*(d*_*xy*_*)* the constant distance decay function applied to the distance *d*_*xy*_ between population location x and provider location *y*. *Prob*_*demand*_ represent the probability of demand according to the Huff Model and *C*_*x*_ is the effective catchment size at population location *x*. The steps necessary to compute AI are similar to the steps of the 2SFCA method explained above.

Implementation of a decay function within the iFCA method has to consider one global parameter: the global catchment size *C*_*glob*_ in which the decay function will have to fit in. In other words, the global catchment size defines the maximum distance (in minutes) up to which distances between population location x and provider location y are considered. We defined the global catchment size from the populations’ point of view and not the providers point of view. Accordingly Ni et al. suggested a constraint for allocating demand and supply: the catchments of both the population (demand) and the provider (supply) must intersect in order to allocate demand and supply [19]. Since potential access and not the actual use of access is measured, defining the catchment size from the populations point of view seems more appropriate. However, the proper choice of a global catchment size still lacks valid empirical data and therefore the choice has to be guided by a theoretical concept depending at least on the respective health service, the country and the mode of transport. For developed countries such as the United Kingdom a maximum catchment size of 60min by car to a GP practice is commonly used. This catchment size is also used by the Office for National Statistics in England.

A commonly used decay function within the FCA methods is the Gaussian function [8,9,13,17,23–26]. The right branch of the Gaussian function used in these studies has a downward S-shaped graph. We wanted to provide this S-shape while increasing flexibility of the function. Therefore, we defined the decay function as a downward sigmoid function (S-shape) following a logistic distribution. The downward log-logistic function as a distance decay function has been show to fit commuter behaviors better than exponential or power functions [27]. However, for reasons to come we used the logistic cumulative distribution function (CDF) of the logistic function instead of the log-logistic function or the Gaussian function. The CDF of the logistic function takes the following general form:

$$CDF(d)\;=\frac{T}{1+e^{\frac{d-\alpha }{\beta }}}$$ (4)

where *T* represents the asymptotic ceiling whereas α and *β* are parameters with *β>0*. The inflecting point is α and represents the median of the function. We chose the median over the mean since the median is less influenced by outliers. The steepness of the function is defined by *β*. For the CDF the variance of the function is defined as follows:

$$Variance\;={SD}^{2}\;=\frac{\beta^{2}*\pi^{2}}{3}$$ (5)

where *SD* is the standard deviation. Therefore, *β* is defined as follows:

$$SD\;=\frac{SD*\sqrt{3}}{\pi }$$ (6)

Eq 4 and Eq 6 combined is shown in the following equation:

$$CDF(d)\;=\frac{T}{1+e^{\frac{(d-median)*\pi }{SD*\sqrt{3}}}}$$ (7)

Through this step the arbitrary value choice of *β* is replaced by the easily calculated SD of the distribution and therefore the steepness of the curve is dependent on a variable rather than a fixed parameter value.

For the FCA-method a condition is f(0) = 1. The implications of this condition are addressed in the discussion in more detail. This condition is fulfilled by adapting the ceiling of the function *T* in Eq 9 so that f(0) = 1.

$$T=1+e^{-\frac{(Median)*\pi }{SD*\sqrt{3}}}\;,\;\text{for f}\left(0\right)\;=1$$ (8)

Therefore, the final adjusted decay function *f*_*adj*_*(d*_*xy*_*)* for the integrated FCA method is:

$$f_{adj}\left(d_{xy}\right)\;=\frac{1+e^{-\frac{(Median)*\pi }{SD*\sqrt{3}}}}{1+e^{\frac{\left(d_{xy}-Median\right)*\pi }{SD*\sqrt{3}}}}$$ (9)

Furthermore, as outlined by Delamater, besides an adjusted decay function *f*_*adj*_*(d*_*xy*_*)*, an additional decay function has to be added to address the shortcoming of container-like systems (*f*_*con*_*(d*_*xy*_*))* [10]. In contrast to the adjusted decay function, the constant distance decay function (*f*_*con*_*(d*_*xy*_*))* only depends on the global catchment size *C*_*glob*_ *and its derived SD (SD*_*glob*_*)*: the median was substituted by *C*_*glob*_/2 and the SD was substituted by *SD*_*glob*_ Therefore, the constant decay function has the following general form:

$$f_{con}\left(d_{xy}\right)\;=\frac{1+e^{-\frac{\left(C_{glob}/2\right)*\pi }{SD_{glob}*\sqrt{3}}}}{1+e^{\frac{\left(d_{xy}-C_{glob}/2\right)*\pi }{{SD}_{glob}*\sqrt{3}}}}$$ (10)

SD_glob_ is calculated for *f*_*con*_*(C*_*glob*_*)* = 0.01 (i.e. at the value of the global catchment size the weight value equals 0.01). This cut off value was reported as a critical value within the Gaussian function approaching zero [16,28]. If *C*_*glob*_ is known, *SD*_*glob*_ can be calculated with the following formula:

$${SD}_{glob}\;=\frac{\pi *\text{ln}\left(e\right)*C_{glob}}{2*\sqrt{3}*\text{ln}(100)}$$ (11)

Both functions are used to model the travel behavior of patients to health service providers dependent on the distance. Due to the nature of the described functions every population location *x* is assigned a differently shaped *f*_*adj*_*(d*_*xy*_*)*, whereas *f*_*con*_*(d*_*xy*_*)* is identical to all population locations.

The combination of both decay functions results in an individual effective catchment size C_x_ for each population location *x*. C_x_ is defined as the distance *d* for which f_adj_(d)*f_con_(d) = 0.01. The global catchment size *C*_*glob*_ defines the maximum distance, which is used to generate the raw data. The effective catchment size C_x_ defines the maximum distance that is used to compute the accessibility Index (AI_x_) at population location *x*. Since the distance d is measured as the travel time on roads depending on road specific speed limits, the shape of the catchment area is dependent on the road network. In a country, where the road network is elaborated the shape of the catchment area is likely to be more or less circular with irregular boundaries. However, in a country with less elaborated road networks the shape of the catchment area could take a variety of forms depending on the road network.

Competing supplier are considered within the Huff Model: The probability of demand from population location *x* on a health service provider at location *y* is dependent on alternative health service providers at other locations *z*, as long as those are within C_x_ of population location *x*.

$$Prob_{demand}=\frac{S_{y}*f_{adj}\left(d_{xy}\right)}{\sum_{z\in (d_{xz}\leq C_{x})}S_{z}*f_{adj}(d_{xz})}$$ (12)

#### Case study

We used the proposed method in a case study measuring the spatial accessibility of primary care physicians in Berlin, Germany. The addresses of primary care physicians located in Berlin were retrieved via the Association of Statutory Health Insurance in Berlin [29]. The geocoding process was done with an application programming interface for Google Maps [30]. The population within the 447 administrative districts of Berlin was retrieved from the Federal Bureau of Statistics of Berlin-Brandenburg as of 2013 [31]. The vector data of these 447 districts were obtained from the Senate Administration for City Development and Environment as of 2015 [32]. The network dataset used, was based on open street map (OSM) data as of 2011 [33]. For the geospatial analyses ArcGIS 10.4 (ESRI Inc, Redlands, CA) with the Network Analyst Extension was used. In addition, further computations were performed with SPSS 23 (IBM, Armonk, NY).

## Results

### Introduction of variable distance decay

**Fig 1:**
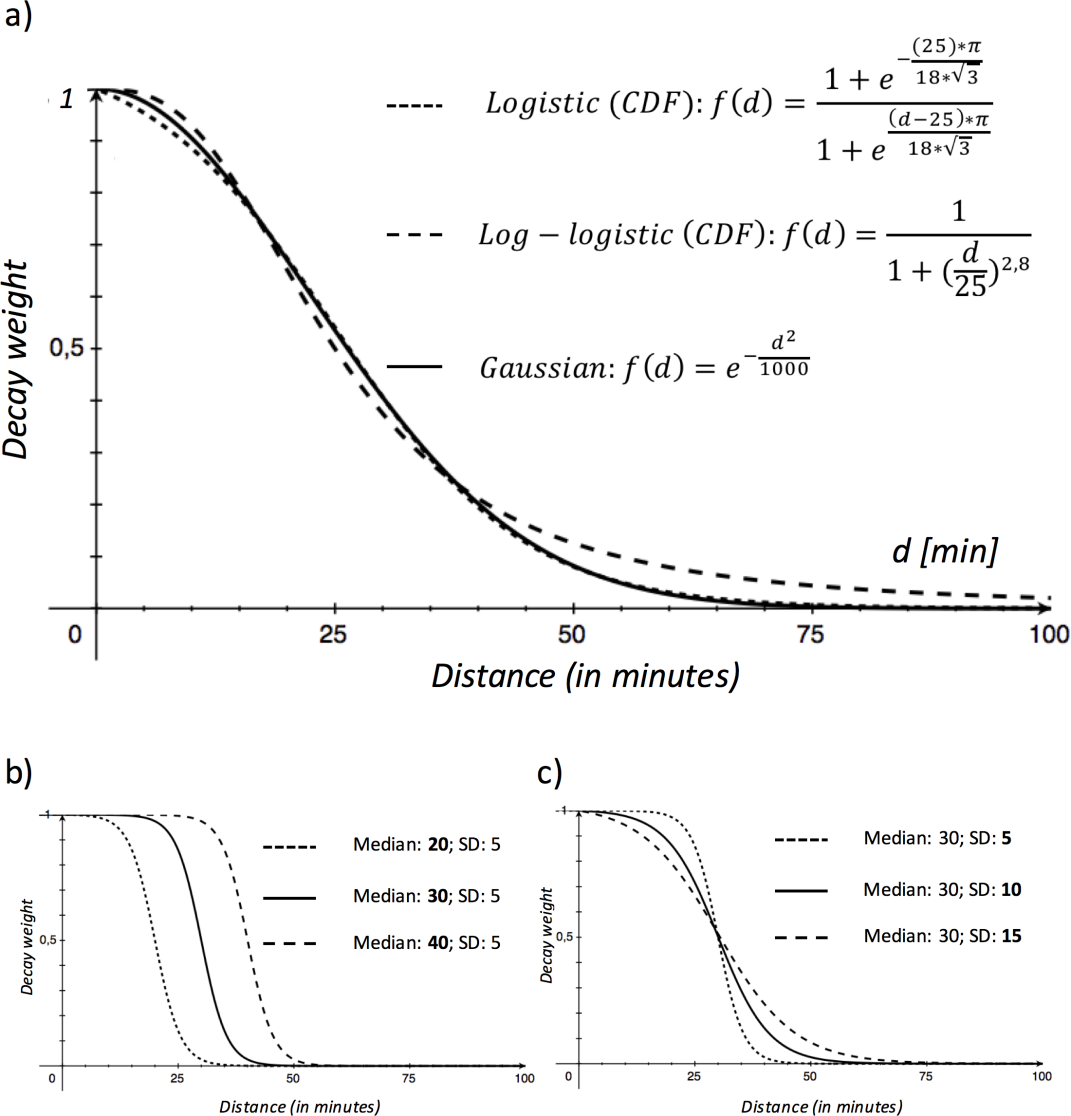
**a) Comparison of the three different decay functions, Gaussian, logistic and log-logistic. b-c) Different configurations of the logistic decay function f_adj_(d).** b) horizontal shift depending on the median and c) steepness depending on the standard deviation (SD) CDF: cumulative distribution function.

We started with a comparison of the proposed logistic distance decay function with commonly used decay functions within the FCA methods: The Gaussian and the log-logistic function (Fig 1A).

Between the Gaussian and the logistic function there is minimal difference in the beginning and almost none in the midsection and the tail of the function. In comparison with the log-logistic function, there are more differences in the beginning as well as in the tail. However, our decay function was built to adapt to every population location *x* by depending on the median and SD of the distribution of population-to-provider distance pairs (within a global catchment size C_glob_). Therefore, every population location *x* has an individually shaped decay function *f*_*adj*_*(d*_*xy*_*)*. Adapting to the median results in a horizontal shift of the function (Fig 1B) and adapting to the SD results in a change of steepness (Fig 1C). In other words, adapting the function to the median distance to provider’s accounts for availability: The greater the median distance to providers, the more likely are patients willing to travel greater distances. Adapting to the SD accounts for agglomeration: The higher the provider agglomeration (i.e. smaller SD), the less likely are patients willing to travel further than the distance to the agglomeration. Therefore, a high provider agglomeration (such as a major city) works as a distance threshold with higher weightings for smaller distances and smaller weightings for greater distances.

### Improvement of catchment parameters

**Fig 2:**
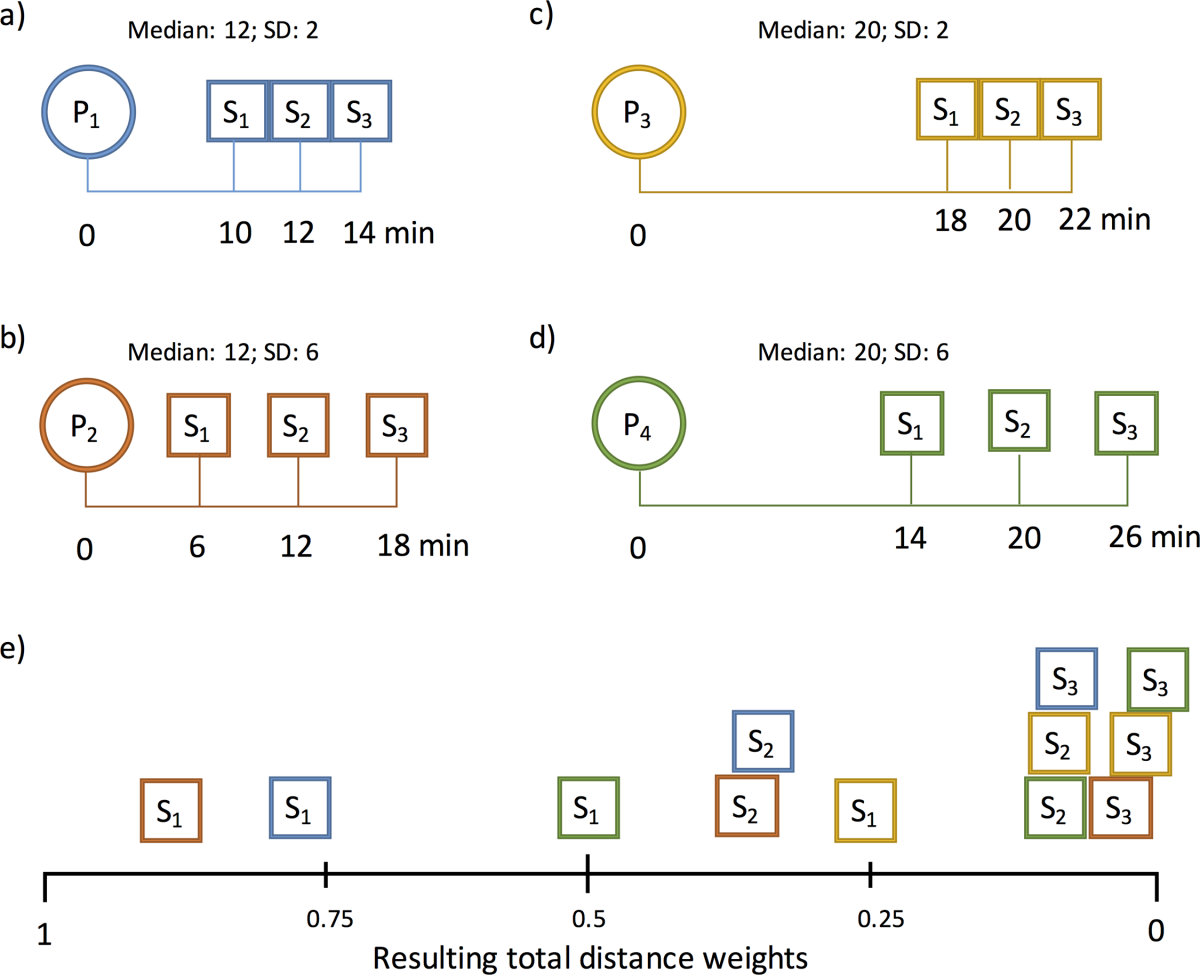
**Four configurations (a-d) of population locations (P_1-4_) and three provider locations (S_1-3_) within a 30min global catchment. e) shows the resulting total distance weight for distances between population and providers (see matching colours).** Distances are for illustration purposes only and hence not true to scale.

We applied the proposed iFCA method within four theoretical examples (Fig 2A–2D) to show the effect of provider locations on the distance decay function. In this theoretical setting there are four different and independent population locations (P_1-4_) within a study area. The global catchment size *C*_*glob*_ is set to 30min. For *f*_*con*_*(30)* the standard deviation SD_glob_ was 5.91. Every population location has three providers (S_1-3_) located within that catchment. However, the configuration of provider locations in every example differed in regard to the median distance and the standard deviation:

- P_1_: Median ↓ and SD ↓
- P_2_: Median ↓ and SD ↑
- P_3_: Median ↑ and SD ↓
- P_4_: Median ↑ and SD ↑

**Fig 3:**
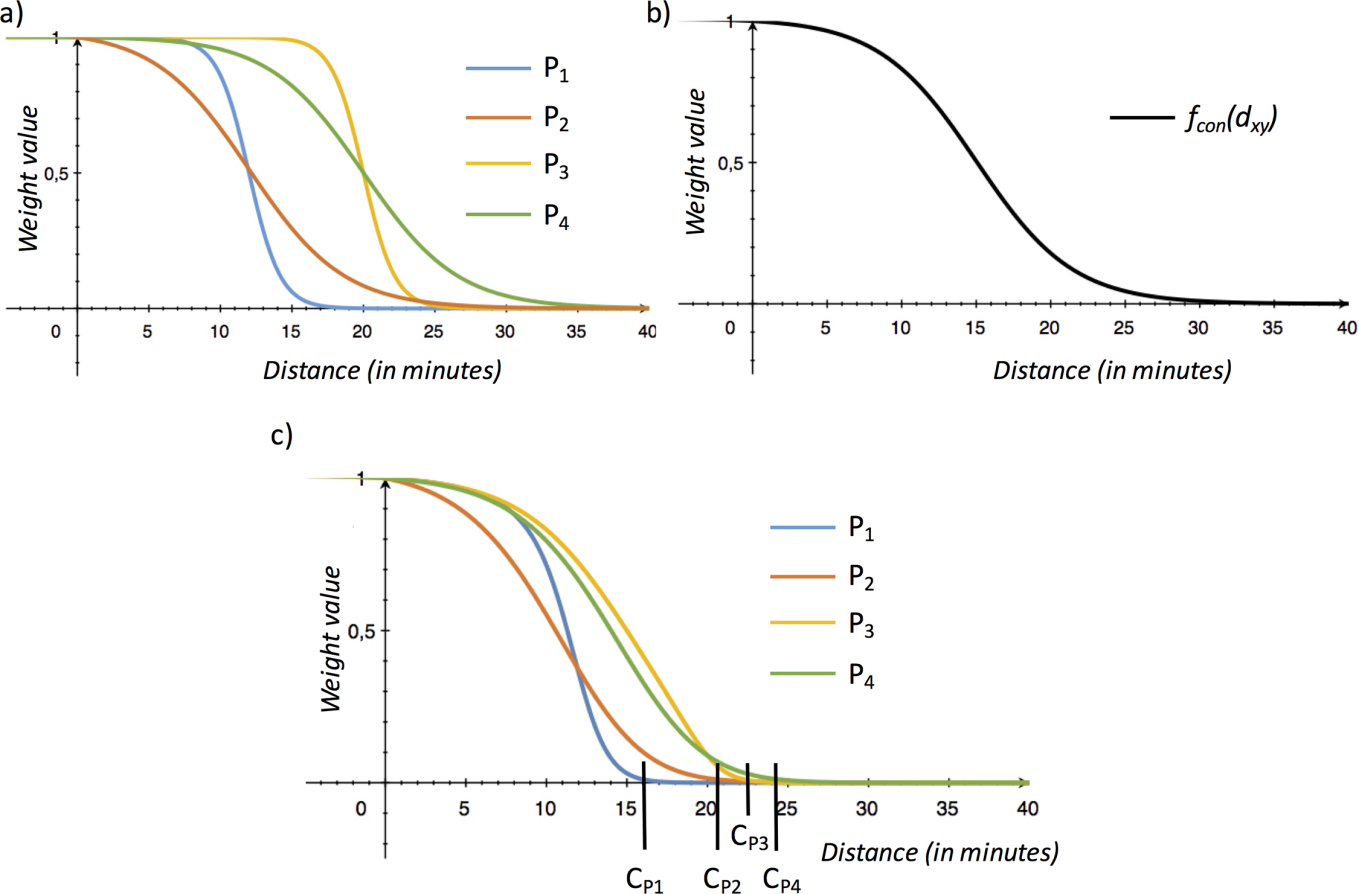
Shape of a) adjusted decay functions *(f*_*adj*_*(d*_*xy*_*)*), b) the constant decay function (*f*_*con*_*(d*_*xy*_*)*) and c) the total distance decay functions (*f*_*adj*_*(d*_*xy*_)* f_*con*_*(d*_*xy*_)) for the four population locations P1-4. C_p1-4_ are the resulting effective catchment sizes.

Applying the adjusted distance function resulted in four differently shaped functions for *f*_*adj*_*(d*_*xy*_*)* (Fig 3A). For the study area (including P_1-4_) the constant decay function *f*_*con*_*(d*_*xy*_*)* is shown in Fig 3B. The adjusted distance functions are shaped according to the median (horizontal shift) and SD (steepness) as outlined in the method section. In addition, and for a better understanding the resulting total distance decay functions (f_adj_(d_xy_) * f_con_(d_xy_)) defining the effective catchment sizes C_x_ is displayed in Fig 3C. This resulted in four different effective catchment sizes (C_P1-4_) according to the total distance weight (Fig 3C):

- C_P1_: 16.09 min
- C_P2_: 20.76 min
- C_P3_: 22.36 min
- C_P4_: 24.61 min

Furthermore, the exact values of the resulting total distance weights are shown in Table 1 and a visual ranking is shown in Fig 2E. Since the adjusted distance function *f*_*adj*_*(d*_*xy*_*)* is individually adjusted to the distribution of provider locations, the weightings among the nearest provider (S_1_), the provider in the middle (S_2_) and the farthermost provider (S_3_) have more or less equal weightings among all population locations (P_1-4_). The constant distance function could be seen as the fixed distance function used in other variations of the FCA methods and thus follows the simple rule: same distance, same weight. Therefore, S_3_ of P_1_ has the same weight (0.5819) as S_1_ of P_4_. According to the effective catchment size C_x_ being defined as *f*_*adj*_*(d*_*xy*_)*f_*con*_*(d*_*xy*_*) = 0*.*01*, the resulting distance weight of S3 and P4 (0.0047) is smaller than 0.01 and therefore, S3 would be neglected in the computation of the accessibility index of P4. This is further evident by the distance value of the effective catchment size of P4 (C_P4_ = 24.61min) in comparison with the distance of P4 to S3 (d = 26min), which is larger than its effective catchment size.

**Table 1:** Exact values of weights according to the adjusted decay functions *f*_*adj*_*(d*_*xy*_*)*, the constant decay function f_con_(d_xy_) and the total distance decay functions *f*_*adj*_*(d*_*xy*_)* f_*con*_*(d*_*xy*_*)*.

		P1	P2	P3	P4
**RELATIVE WEIGHTS (F**_**ADJ**_**)**	**S1**	0.8598	0.8827	0.8598	0.8619
	**S2**	0.5000	0.5133	0.500	0.5012
	**S3**	0.1402	0.1439	0.1402	0.1406
**ABSOLUTE WEIGHTS (F**_**CON**_**)**	**S1**	0.8309	0.9500	0.2877	0.5819
	**S2**	0.7224	0.7224	0.1791	0.1791
	**S3**	0.5819	0.2877	0.1055	0.0334
**TOTAL DISTANCE WEIGHTS**	**S1**	0.7144	0.8386	0.2473	0.5015
	**S2**	0.3612	0.3708	0.0896	0.0898
	**S3**	0.0816	0.0414	0.0148	0.0047

### Estimation of catchments in Berlin

Since the case study in Berlin was only used for demonstrating the proposed method, demand and supply outside of city boundaries were neglected. Therefore, the presented results do not reflect realistic potential access. Within Berlin n = 2,382 primary care physicians were located in 2013. The total population size was n = 3,517,424 located within n = 447 administrative districts. Taking 447 population centroids as origins (O), locations of the 2,382 primary care physicians as destinations (D) and a global catchment size of 30min as input data, resulted in n = 976,863 OD pairs. For *f*_*con*_*(d)* the standard deviation for which f(30) = 0.01 was SD_glob_≈5.91. The metrics of the resulting OD pairs for three different catchment sizes are shown in Table 2.

**Table 2:** Metrics of the integrated FCA method for all n = 447 population locations for three global catchment sizes. In addition, metrics are shown for population locations ‘example 1’ and ‘example 2’.

		*AI*	*Median of OD distance (min)*	*SD of OD distances (min)*	*effective Catchment (min)*	*OD Pairs with f*_adj_*f_*con*_*≥0*.*5*
**C_glob_ = 15min**	Min	0.000	7.51	2.00	11.3	1
	Max	0.500	14.38	5.17	14.1	583
	Mean	0.237	10.54	3.39	12.6	249
	Example 1	0.140	12.39	3.85	13.4	125
	Example 2	0.421	8.92	3.56	12.0	500
**C_glob_ = 30min**	Min	0.421	9.49	3.96	19.1	11
	Max	0.959	26.86	6.91	27.3	1099
	Mean	0.667	16.30	6.00	22.9	650
	Example 1	0.734	18.23	6.03	23.8	437
	Example 2	0.804	11.41	5.75	20.3	1011
**C_glob_ = 45min**	Min	0.493	9.35	5.66	22.0	171
	Max	1.236	32.04	8.45	37.1	1187
	Mean	1.010	16.43	6.86	28.1	963
	Example 1	1.187	19.52	7.85	31.0	900
	Example 2	0.976	11.08	5.85	23.4	1147

We will discuss the results regarding a global catchment size of 30min in more detail: These data showed greatly varying medians within the study area. Also the SD’s varied, however to a smaller extent. This corresponded to variable distance decay functions, which led to effective catchment sizes between 19.1–27.3 min. The effective catchment sizes caused 159,567 OD pairs (16.3%) to have total distance weights of less than 0.01 and therefore were not included in the computation of the accessibility index.

**Fig 4:**
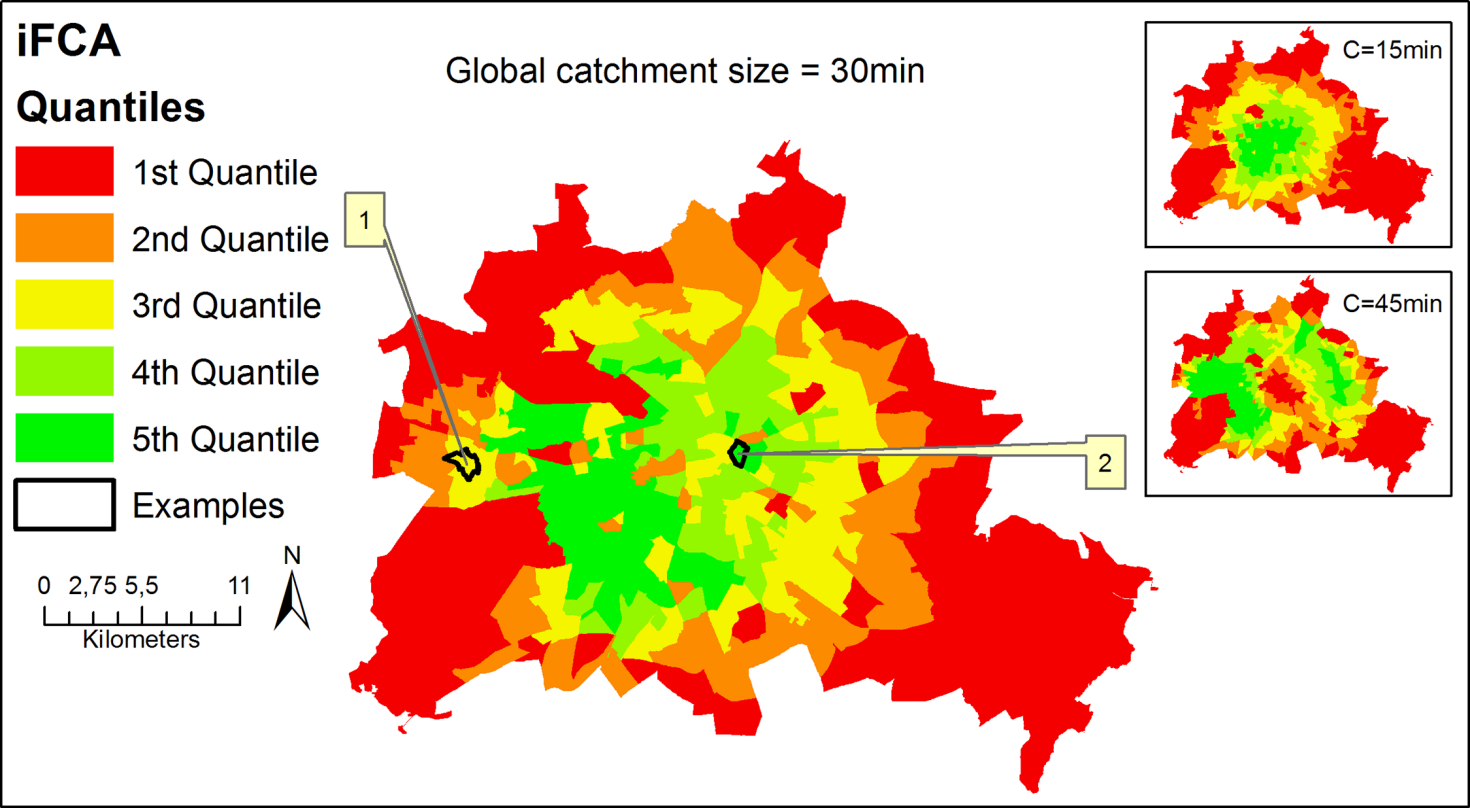
Integrated FCA method applied to Berlin with a global catchment size of 30min. **In addition, results are shown for a global catchment size of 15min and 45min.** Examples see text. This figure is a derivative of " RBS-LOR, Lebensweltlich orientierte Räume, Dezember 2015” by “Amt für Statistik Berlin-Brandenburg” used under CC BY 3.0 DE.

The results of the iFCA method are shown in Fig 4 and Table 2. The city center appeared to have a higher spatial accessibility than some clusters outside of the city center. For demonstrating purposes, two selected population locations, example 1 and 2 in Fig 4, will be examined in more detail to understand the displayed pattern.

Example 1 (official name ‘Planungsraum Adamstraße’) is taken from a pattern with low accessibility indices in the Midwest of Berlin. Example 2 (official name ‘Planungsraum Karl-Marx-Allee’) is taken from a pattern with rather high accessibility indices in the center of Berlin.

**Fig 5:**
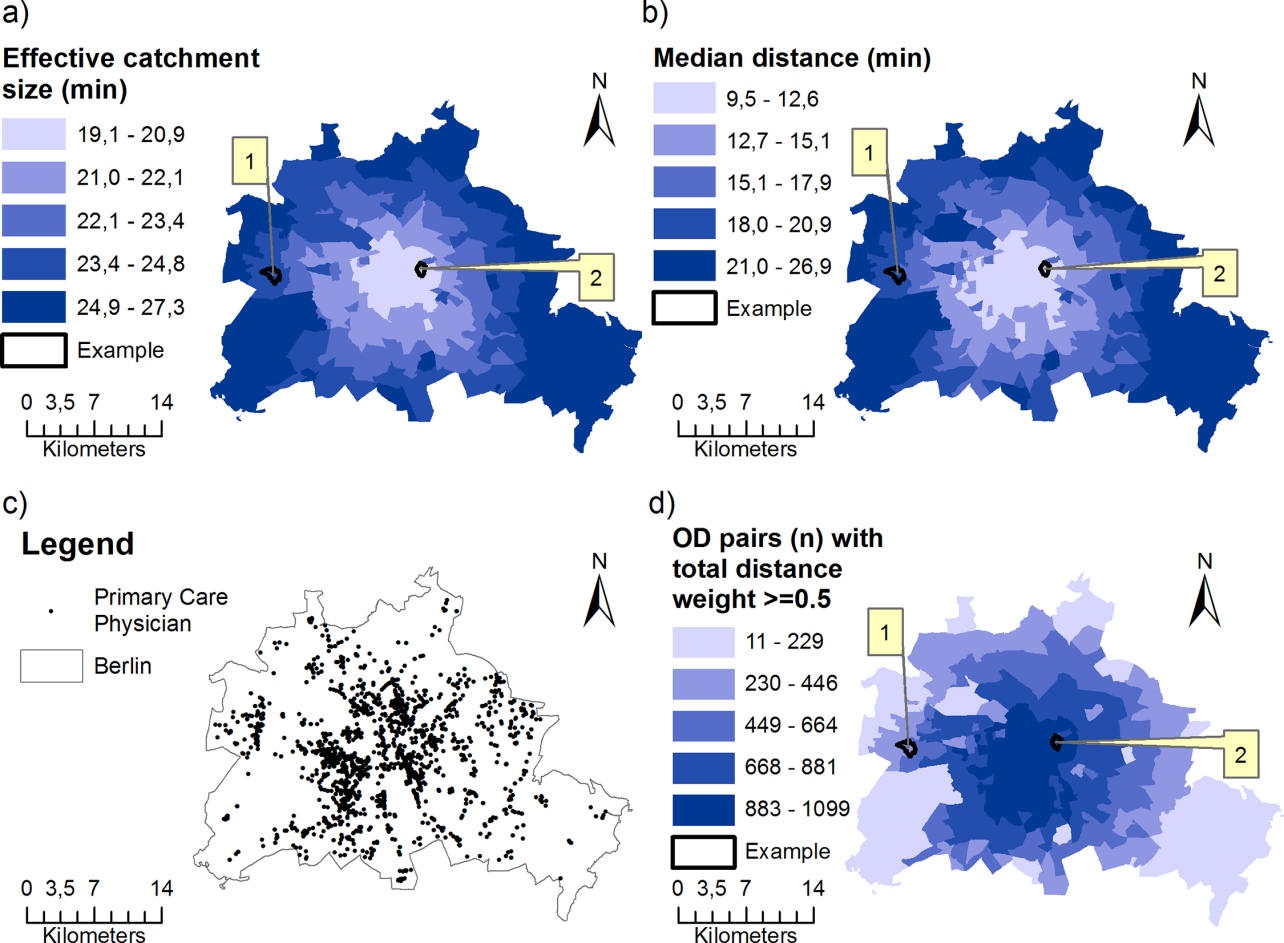
Geographical distribution of parameters of the integrated FCA method (for a global catchment size C_glob_ = 30min). This figure is a derivative of " RBS-LOR, Lebensweltlich orientierte Räume, Dezember 2015” by “Amt für Statistik Berlin-Brandenburg” used under CC BY 3.0 DE.

In order to identify the cause for the index patterns, we will demonstrate the distribution pattern of parts compiling the iFCA measurement (Eq 3). Recapitulation of the iFCA equation shows that the accessibility index increases with higher supply (numerator) and lower demand (denominator). Therefore, the index increases if (1) the number (influenced by the effective catchment size) and capacity (*S*_*y*_) of providers increase and (2) distance decay weights increase. However, the capacity *S*_*y*_ in our case study is constant (*S*_*y*_ = 1) since we used the headcount of physicians and can therefore be neglected as an influencing parameter. On the other hand, the index decreases with bigger population sizes (*P*_*x*_) and/or higher probability of demand (*Prob*_*demand*_). In addition, the geographical distribution of some key parameters is shown in Fig 5.

The lower access index of example 1 in comparison to example 2 was mainly due to a greater median and SD, which led to a larger effective catchment size (Fig 5A and 5B). Furthermore, the number of OD pairs with distance decay scores **≥** 0.5 (Fig 5D) was higher for example 2 than 1.

Furthermore, we analyzed the behavior of the distance decay for differing global catchments: A global catchment of C_glob_ = 15min resulted in n = 447,828 OD pairs with a mean effective catchment size of 12.6min and a global catchment of C_glob_ = 45min resulted in n = 1,060,776 OD pairs with a mean effective catchment size of 28.1min. The effect of differing catchment sizes on the accessibility index is shown in Table 2: For C_glob_ = 15min the accessibility index was lower for example 1 than example 2. However, for C_glob_ = 45min the accessibility index was higher for example 1 than example 2. This finding emphasizes the importance of an adequate parameter choice of the catchment sizes.

**Fig 6:**
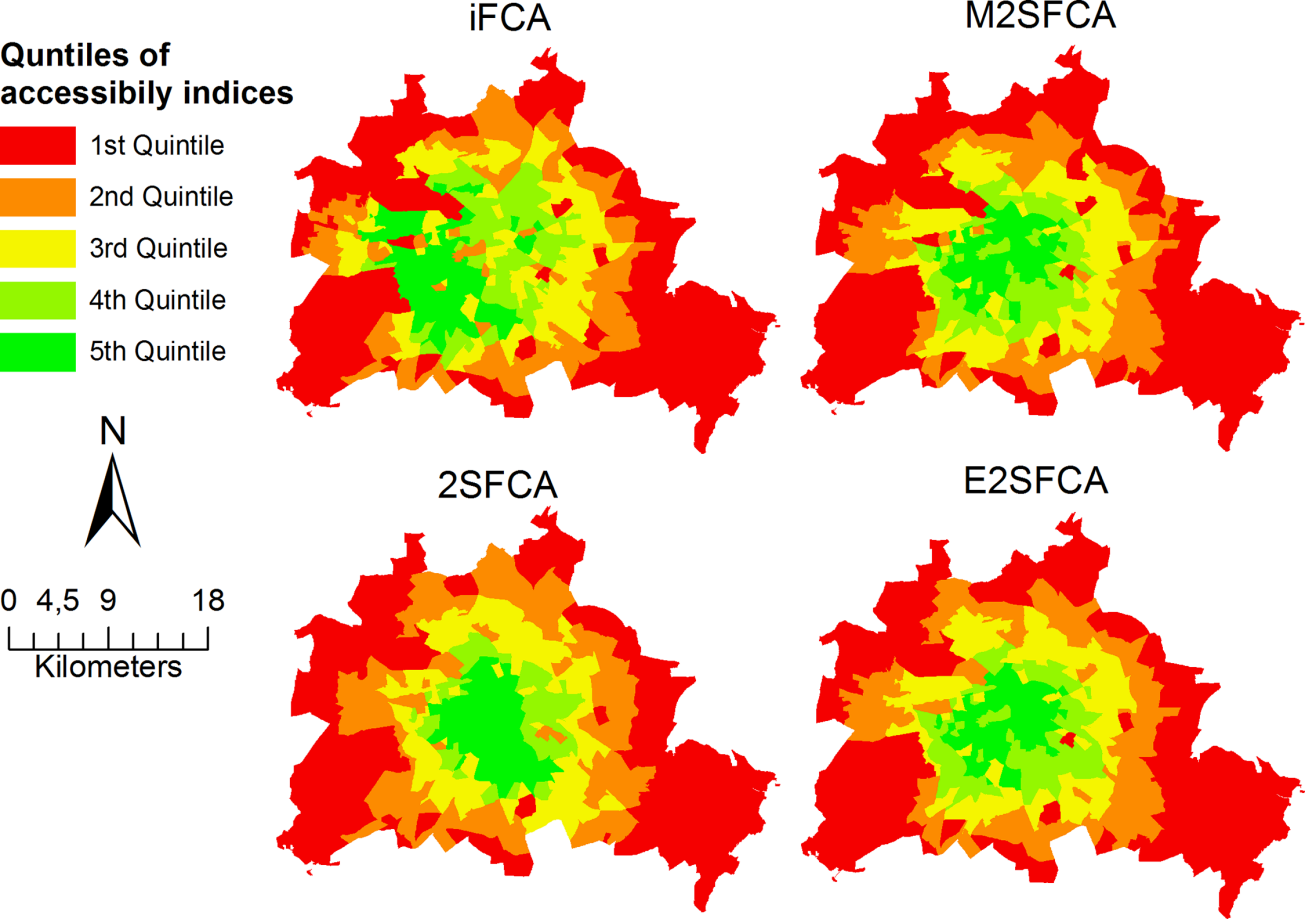
Results of the iFCA, 2SFCA, E2SFCA and the M2SFCA method applied to the study area of Berlin. This figure is a derivative of " RBS-LOR, Lebensweltlich orientierte Räume, Dezember 2015” by “Amt für Statistik Berlin-Brandenburg” used under CC BY 3.0 DE.

Lastly, for benchmark purposes, the iFCA method was compared with the 2SFCA method, the E2SFCA method and the M2SFCA method. The E2SFCA method used a Gaussian decay function with a sharp decay equal to three weightings (1.00, 0.42, 0.09) according to three travel zone (0–10,10–20,20–30 min). The M2SFCA method used the downward log-logistic function with empirical tested coefficients: f(d) = 1/(1+ (d/13.89)^1.89^), whereas the 2SFCA did not use an distance decay. For all three methods a global catchments size of 30min was chosen. The results are shown in Fig 6.

The E2SFCA method (Fig 6A) resulted in higher access score in the center and lower near the borders. The M2SFCA method and the 2SFCA also resulted in mostly high accessibility indices near the city center. However, despite using the same measurement concept, all three methods incorporate different parameters and are therefore not directly comparable in this application. Still, due to their same principal concept they were significantly correlated with the proposed iFCA method with r = 0.78 (2SFCA; p<0.001), r = 0.89 (E2SFCA; p<0.001) and r = 0.91 (M2SFCA; p<0.001). Detailed results are provided within a supporting information file (S1 Table).

## Discussion

With this paper we integrated suggested improvements regarding the shortcomings of the crude 2SFCA method into an integrated FCA method ‘iFCA’. To our knowledge these improvements have not been composed to the presented equation (Eq 3). Besides the general form of the equation, we introduced a variable distance decay function within the iFCA method. In the FCA literature however, numerous different functions and values of the impedance coefficient *β* have been used. However, mostly the Gaussian function has been used in combination with 3–5 different catchment zones [9,17,23,25,26]. Still, even if the Gaussian function was agreed upon, the term ‘Gaussian function’ applied to a range of functions depending on the parameter choice. Especially the choice of the impedance coefficient *β* varied in the literature. Therefore, various ‘Gaussian functions’ have been used: for example f(d) = e^-((d-1)^2)/β^ with *β* = 1.15 [17] or with *β* = 1.5 and β = 2.0 [25]. Luo et al. and Wan et al. used f(d) = e^-(d^2)/β^ with *β* = 440 up to *β* = 1040 [8,26].

These examples show that no single best function has been supported so far by the literature even within a subgroup of functions. However, regardless of the chosen function, only constant functions have been used. And since the Gaussian function cannot be shifted as needed due to its bell-shape, we followed the approach of previous research who used logistic based functions [27,34]. We aimed at omitting arbitrary parameter choices (mainly the impedance coefficient β) within the decay function: Both the theoretical and real world application could demonstrate that our distance decay function is only depending on parameters generated by the distribution of providers (i.e. median and SD). Using a dynamic decay function has several benefits. Adapting to the median accounts for availability: The greater the median distance to all providers within the global catchment, the more likely should patients be willing to travel longer distances. Adapting to the standard deviation accounts for agglomeration: The higher the providers’ agglomeration (i.e. smaller standard deviation), the less likely should patients be willing to travel longer distances than the distance to the agglomeration. In other words, the greater the agglomeration, the more the median distance works as a threshold. It has to be noted that S-shaped decay functions (as used in our study as well as in the vast majority of literature) assume up to 100% probability of access if the distance approaches zero. Therefore, the cumulative probability will by far exceed 100%, especially if an agglomeration occurs (small SD). However, since potential access is measured, this issue is less relevant compared to the measurement of the actual use of access, where the cumulative probability must not exceed 100%.

By individually adapting the shape of the decay function, another shortcoming is accounted for: variable catchment sizes. Variable catchment sizes within the global catchment size are effectively implemented by the asymptotic approach of weight values to zero. These catchment sizes are referred to as ‘effective catchment sizes’ in contrast to the ‘global catchment size. The need for variable catchment sizes has been empirically demonstrated in a recent survey among 1,079 study subjects: the maximum tolerable travel distance varied significantly between rural and urban areas (54.1 vs 31.9 min, p<0.001) [35]. Therefore, differing travel behaviors depending on location can be assumed and choosing equal catchment sizes especially for small scale analyses must be considered inappropriate. We showed that our variable distance decay function effectively influenced the effective catchment size within the global catchment size and therefore fulfilled the need for variable catchment sizes without having to pre-determine variable catchment sizes as in earlier approaches [17–19,35].

Still the presented method is far from being complete. Several factors must be considered additionally. For example the travel mode (car, bike, public transport) has an major impact on the accessibility [36]. Therefore, Langford et al. proposed a multimodal approach to account for differing modes of transport [37]. Furthermore commuting behavior has a potential impact [38].

The choice of the global catchment size remains arbitrary due to lacking empirical data. In our case study we chose 15, 30 and 45 min for primary care physicians in an urban setting which is in line with current literature [17,25,26]. However, other medical specialties and countries most likely need adjustment of the global catchment size: Wan et al. for example chose 180min for oncologists [26]. As supported by our analysis of the three different global catchment sizes, the effect of an adequate catchment size choice is crucial to the outcome of the measurement.

It should be emphasized that the best way to choose an appropriate distance decay function is the empirical validation of a certain function for a certain setting. However, since practical limitations across different settings makes the empirical validation difficult, our suggested function with its variable parameters can adapt to a variety of settings. Still, empirical data are crucial to promote one function over the others. Therefore future research should focus on the validation of different decay function within different settings.

The comparison of the proposed iFCA method with the crude 2SFCA method, the more established E2SFCA method and the also fairly new MS2FCA method showed minor differing results. Other studies have also shown more similar results of the methods [10]. Most likely these differing results are due to (1) the large-scale application and (2) provider choice. Other studies have used the methods in rather small-scale applications (i.e. larger areas) and also with hospitals as opposed to primary care physician used in this study [10,26]. Furthermore, since additional interfering variables have been introduced to a different extent in the methods, differing results are likely to occur and comparison of the methods is limited.

### Conclusion

For the first time, we introduced a variable distance decay function within the FCA methods. The functions’ shape is set by generated distribution data only and in addition provides effective variable catchment sizes. Furthermore, the proposed integrated FCA method integrates recent improvements on shortcomings regarding earlier FCA-methods and therefore takes relevant influencing factors into account. A case study demonstrated the general fit of the proposed method.

## Supporting Information

S1 Appendix Review of FCA shortcomings. (DOCX)

S1 Table Detailed results of spatial accessibility for administrative areas in Berlin (n = 449). (XLSX)
