# Supplementary material for: Measuring Spatial Accessibility of Health Care Providers – Introduction of a Variable Distance Decay Function within the Floating Catchment Area (FCA) Method
Source: PLoS One. 2016 Jul 8;11(7):e0159148. doi: 10.1371/journal.pone.0159148 (PMC4938577; doi:10.1371/journal.pone.0159148)
Supplement: S1 Appendix — (DOCX) [file pone.0159148.s001.docx]

**Review of FCA shortcomings**

**Shortcoming ‘distance decay’**

Luo et al. developed the enhanced (E)2SFCA method by dividing the catchment area into three zones and applying a discrete Gaussian function as the decay function [1]. Instead of a discrete Gaussian function, Dai used a continuous Gaussian function, respectively a continuous kernel density function (KD)2SFCA [2,3]. Wang reported six functions used within the 2SFCA methods: 1) gravity function, 2) Gaussian function, 3) binary discrete, 4) multiple discrete, 5) kernel density and 6) three zone hybrid [4]. Recently also logistic based functions have been used within the FCA methods [5]. The following continuous decay functions are commonly used [6–8]:

- Gravity function: f(d)=d^-β^
- Exponential function: f(d) = e^-β*d^
- Inverse power function: f(d) = e^-β^
- Gaussian function: f(d) = e^-(d^2)/β^
- Logistic based function: f(d)= 1/(1+e ^- β (d- mean)^ )

Since the 2SFCA method is based on a gravity model, it has been shown that the overall system has container like properties meaning the overall outcome does not change if supply or demand locations change [9]. Therefore, the 2SFCA method is blind to configurations within the system, which is caused by a balance between the two distance decay functions, which are added to the numerator as well as the denominator. To address this shortcoming, Delamater et al. developed the modified (M)2SFCA method by adding an second distance decay function to the formulae [9].

However, due to a lack of data there is still uncertainty over the appropriate function to choose. Besides the selection of the function itself, the choice of the appropriate parameter, namely the impedance coefficient *β* further increases uncertainty [10]. As pointed out by Wang the *β* parameter itself should rather be a variable instead of a constant [11]. So far, the *β* parameter has only been used as a constant with varying values in different studies [8,11–13].

### Shortcoming ‘catchment size’

Conceptualizing catchment areas is difficult, since there is a lack of reliable empirical data describing the distribution, composition and profile of patients who access health services [14]. Therefore, catchment sizes in the literature are described in a wide range of diversity, e.g. depending on the mode of transport (walking vs. automobile vs. public transport) or the concept of distance (minutes vs. kilometers).

However, regardless of the specific configuration of the catchment size, the literature suggests that any catchment size used in a scientific approach to measure access should be variable: Luo et al. developed the variable (V)2SFCA method by implementing variable catchment sizes [15]. They determined the catchment size by incrementally increasing catchment sizes until a defined base population respectively a defined PPR is reached. A similar approach was used by Ni et al., who used a threshold ratio instead of an absolute base population number and called their improved method enhanced variable (EV)2SFCA method [13]. Also McGrail et al. suggested dynamic catchment sizes [12].

### Shortcoming ‘competition’

In order to account for competition in the demand-supply system of healthcare, Wan et al. integrated an additional weighting variable based on competition by accounting for the number of competitors within a catchment [16]. The additional first step in this method has led the authors to the name this variation “3SFCA” method. A similar approach was used by Luo et al. by integrating the Huff Model into the 2SFCA method [8]. The Huff Model was first developed by Daniel Huff and can be used to adjust probability of demand by taking alternative competing supplier into account [17]. Li et al. proposed to incorporate an optimization model, which accounts for congestion [18]. However, integrating the Huff model for the demand has comparable effects as the optimization model.

Literature:

1. Luo W, Qi Y. An enhanced two-step floating catchment area (E2SFCA) method for measuring spatial accessibility to primary care physicians. Health Place. Elsevier; 2009;15: 1100–1107. doi:10.1016/j.healthplace.2009.06.002

2. Dai D. Black residential segregation, disparities in spatial access to health care facilities, and late-stage breast cancer diagnosis in metropolitan Detroit. Health Place. Elsevier; 2010;16: 1038–1052. doi:10.1016/j.healthplace.2010.06.012

3. Dai D, Wang F. Geographic disparities in accessibility to food stores in southwest Mississippi. Env Plann B Plann Des. 2011;38: 659–677.

4. Wang F. Measurement, Optimization, and Impact of Health Care Accessibility: A Methodological Review. Ann Assoc Am Geogr. 2012;102: 1104–1112. doi:10.1080/00045608.2012.657146

5. Delamater PL, Messina JP, Grady SC, WinklerPrins V, Shortridge AM. Do More Hospital Beds Lead to Higher Hospitalization Rates? A Spatial Examination of Roemer’s Law. PLoS One. 2013;8: e54900. doi:10.1371/journal.pone.0054900

6. Kwan M. Space-time and integral measures of individual accessibility: a comparative analysis using a Point-based framework. Geogr Anal. 1998;30: 191–216.

7. de Vries JJ, Nijkamp P, Rietveld P. Exponential or power distance-decay for commuting? An alternative specification. Env Plan A. 2009;41: 461–480. doi:10.1068/a39369

8. Luo J. Integrating the Huff Model and Floating Catchment Area Methods to Analyze Spatial Access to Healthcare Services. T GIS. 2014;18: 436–448. doi:10.1111/tgis.12096

9. Delamater PL. Spatial accessibility in suboptimally configured health care systems: A modified two-step floating catchment area (M2SFCA) metric. Health Place. 2013;24: 30–43. doi:10.1016/j.healthplace.2013.07.012

10. Wan N, Zhan FB, Lu Y, Tiefenbacher JP. Access to healthcare and disparities in colorectal cancer survival in Texas. Health Place. Elsevier; 2012;18: 321–329. doi:10.1016/j.healthplace.2011.10.007

11. Wang L. Immigration, ethnicity, and accessibility to culturally diverse family physicians. Health Place. 2007;13: 656–671. doi:10.1016/j.healthplace.2006.10.001

12. McGrail MR, Humphreys JS. Measuring spatial accessibility to primary health care services: Utilising dynamic catchment sizes. Appl Geogr. 2014;54: 182–188. doi:10.1016/j.apgeog.2014.08.005

13. Ni J, Wang J, Rui Y, Qian T, Wang J. An Enhanced Variable Two-Step Floating Catchment Area Method for Measuring Spatial Accessibility to Residential Care Facilities in Nanjing. Int J Env Res Pub Health. 2015;12: 14490–14504. doi:10.3390/ijerph121114490

14. Allan DP. Catchments of general practice in different countries--a literature review. Int J Health Geogr. 2014;13: 32. doi:10.1186/1476-072X-13-32

15. Luo W, Whippo T. Variable catchment sizes for the two-step floating catchment area (2SFCA) method. Health Place. 2012;18: 789–795. doi:10.1016/j.healthplace.2012.04.002

16. Wan N, Zou B, Sternberg T. A three-step floating catchment area method for analyzing spatial access to health services. Int J Geogr Inf Sci. 2012;26: 1073–1089. doi:10.1080/13658816.2011.624987

17. Huff D. Defining and Estimating a Trading Area. J Mark. 1964;28: 34–38.

18. Li Z, Serban N, Swann JL. An optimization framework for measuring spatial access over healthcare networks. BMC Health Serv Res. 2015;15: 273. doi:10.1186/s12913-015-0919-8
